# Synthesis of Vertically Standing MoS_2_ Triangles on SiC

**DOI:** 10.1038/srep31980

**Published:** 2016-08-23

**Authors:** Feifei Lan, Zhanping Lai, Yongkuan Xu, Hongjuan Cheng, Zaien Wang, Chengjun Qi, Jianli Chen, Song Zhang

**Affiliations:** 1 Electronic Material Research Institute of Tianjin, Tianjin 300220, P. R. China

## Abstract

Layered material MoS_2_ has been attracting much attention due to its excellent electronical properties and catalytic property. Here we report the synthesis of vertically standing MoS_2_ triangles on silicon carbon(SiC), through a rapid sulfidation process. Such edge-terminated films are metastable structures of MoS_2_, which may find applications in FinFETs and catalytic reactions. We have confirmed the catalytic property in a hydrogen evolution reaction(HER). The Tafel slope is about 54mV/decade.

In the past few years, transition metal dichalcogenides(TMD) have attracted great attention for their considerable potential applications in the fields of catalysis, microeletronics, optoelectronic devices on both conventional and flexible substrates^1^,^2^,^3^,^4^,^5^,^6^,^7^,^8^,^9^. Lots of efforts have been made to realize the applications of TMD. But almost all of these works that have been done are based on the platelet-like morphology on the surface of different substrates. In contrast, a few works have been done to use the edges of these layered materials. In fact, vertically standing TMD materials hold high aspect ratio and dangling bonds^10^. For MoS_2_, the exposed edges means the S dangling bonds. It is the active sites for catalytic reactions. As a layered material, MoS_2_ usually expose the basal planes (it means the flat triangles with the Mo atom terminating surface) as the terminating surface with minimal roughness and dangling bonds. But in the condition of vertically standing MoS_2_, the edges sites were exposed maximally. The exposed edges with high chemical active and may play an important role in many catalytic reactions, such as hydrogen production^11^,^12^,^13^,^14^,^15^, photocatalysis^16^, hydrogen evolution reaction(HER), hydrodesulfurization catalyst used for removing sulfur compounds from oil^17^,^18^,^19^,^20^,^21^,^22^,^23^. In addition, these vertical structures of TMD are ideal channel materials for FinFET^24^. However, edges are usually the rare surface sites of layered materials due to their inherently high surface energy. Increasing the edge dimension is therefore challenging.

## Methods

In this work, we develop a rapid sulfidation process through a large carrier gas flow rate on SiC using a CVD method. The MoS_2_ triangles are aligned vertically to the surface of the substrate. Various characterizations techniques were used to have a good understand to the mechanism of the vertically standing triangles. Furthermore, HER performance of vertically standing MoS_2_ triangles was researched.

**Figure 1:**
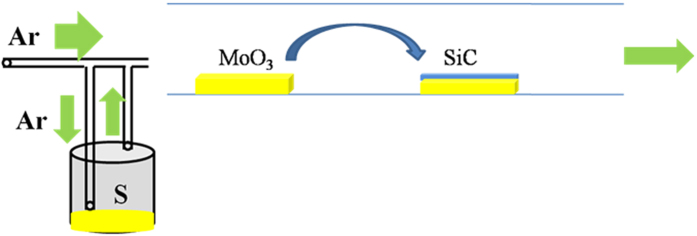
Schematic illustration of the synthesis of vertically standing triangles on SiC.

The synthesis process of vertically standing MoS_2_ triangles is schematically illustrated in Fig. 1. At the beginning, MoO_3_ powder was placed in the centre of the furnace, 6H SiC was placed next to the MoO_3_ powder. Sulphur powder was placed inside of a steel cylinder out side of the furnace, with a heating tape around it. High pure argon was chosen as carrier gas to convey sulphur and MoO_3_ vapor to downstream. The temperature of MoO_3_ powder was 1000 °C, sulphur powder was heated to 260 °C, the growth pressure was atmospheric.

**Figure 2:**
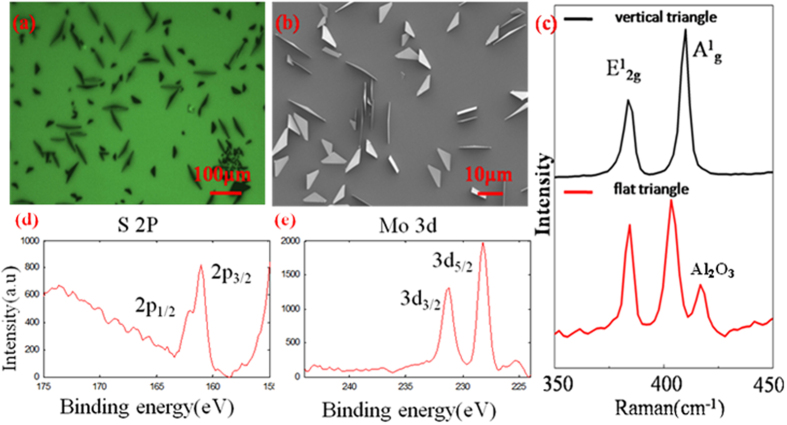
Characterization of synthesized vertically standing MoS_2_ triangles. (**a**) optical image of vertically standing triangles. (**b**) SEM image to clearly shows the vertically standing triangles. (**c**) Raman spectrum of the triangles on SiC and single-layer MoS_2_ obtained by CVD on sapphire. (**d**) XPS spectra of Mo 3d. (**e**) XPS spectra of S2p peak.

A typical optical image of the MoS_2_ triangles grown on SiC substrate is shown in Fig. 2a. It clearly shows that most of the triangles on the surface were vertically standing, there are a few flat triangles on the surface. The SEM image demonstrates that the as grown triangles are nearly perpendicular to the substrate. There is a small angles of inclination of some triangles. The edges of different MoS_2_ triangles could be clearly observed in SEM images. The lateral dimensions of the triangles are tens micrometer, the heights are from 30 nm to 2 μm. Figure 2c is the Raman spectrum of the vertically standing triangles on SiC and monolayer MoS_2_ on sapphire. Two Raman characteristic bands of vertically standing triangles at 410 cm^−1^ and 383 cm^−1^ corresponding to A^1^_g_ and E^1^_2g_ respectively^25^,^26^,^27^. Comparing to the flat monolayer MoS_2_ on sapphire, the intensity ratio between A^1^_g_ and E^1^_2g_ of the vertically standing triangles is higher, revealing a higher density of exposed edges in those vertically standing triangles. Figure 2d,e are the XPS spectra of Mo 3d and S 2p peak. The Mo 3d shows two peaks at 232.5 eV and 229.2 eV. The peaks, corresponding to the S 2p1/2 and S 2p3/2 orbital of divalent sulfide ions (S^2−^), are observed at 163.3 and 162 eV. These results agree well with the reported values for MoS2 crystal^23^,^28^,^29^.

Due to the anisotropic bonding and the general tendency to minimize the surface energy, nanoparticles of layer materials usually exhibit platelet-like morphology^30^,^31^. Alternatively, vertically standing triangles can also be obtained by a fast growth process^32^, the synthesis rate is mainly affected by the diffusion of product gas on the surface of the substrate. By controlling the reactant concentration, we can obtain the MoS_2_ films with different morphologies. Through regulating the carrier gas flow rate, the sulfidation rate of MoO_3_ can be controlled well. In addition, by changing the carrier gas flow rate, we can have a good understand of the growth process of MoS_2_ film.

**Figure 3:**
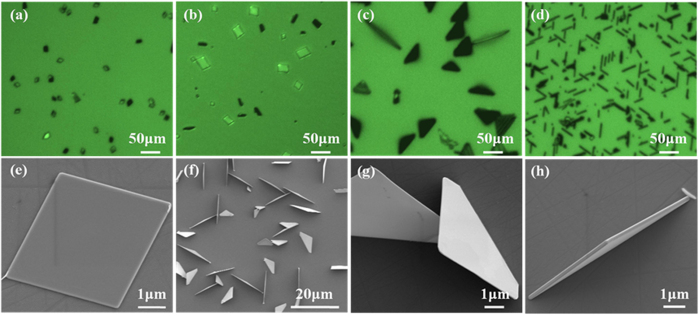
Optical and SEM images of MoS_2_ triangles. (**a–d**) optical images of the MoS_2_ triangles with different carrier gas flow rates. (**e**) SEM image of MoS_2_ rectangles with a low condition of sulfur. (**f–h**) SEM images of vertically standing MoS_2_ triangles.

As shown in Fig. 3a, when the carrier gas flow rate is 100sccm, the concentration of sulfidation vapor is too low to meet the needs. Meanwhile, with a slow carrier gas flow rate, the forming of MoO_3−x_ is limited, and the transport rate of MoO_3−x_ to the surface of substrate is also affected. In this condition, there are only some nanoparticles and some small rectangles on the surface of SiC, because of the lacking of S. So we increasing the carrier gas flow rate to 180sccm, the size of the rectangles increased, but we still do not obtained the vertically standing triangles. When the carrier gas flow increased to 260sccm, the synthesis rate is faster with the increasing of sulfidation concentration. Under this condition, we obtained the vertically standing triangles on the surface of SiC, the result is shown in Fig. 3c. Some of flat triangles were observed on the surface of SiC, only a few vertically standing triangles were obtained. So we increased the carrier gas flow to 340sccm, almost all of the triangles are perpendicular to the substrate, as shown in Fig. 3d. Figure 3e,f were the SEM images of flat rectangles and triangles. Figure 3f–h show the SEM images of vertically standing triangles. From the results, we can see that, only under a fast growth rate, can we get the vertically standing triangles of MoS_2_.

**Figure 4:**
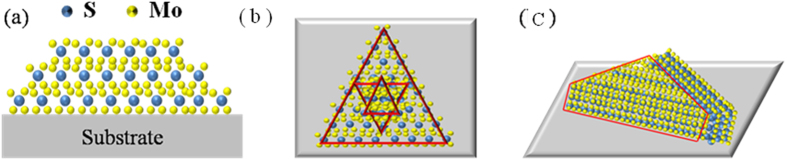
Growth model of vertically standing and flat MoS_2_ triangles.

To have a better understand, the growth model is shown in Fig. 4. At the beginning of the growth, there was neither buffer layer nor seed on the surface of the substrate, so nucleation process was a 3D. The triangles are all coming from the islands of Fig. 4a. When the carrier gas flow rate is small, the atoms and molecules have enough time to mobility and diffusion on the surface of the substrate, so the synthesis process of MoS_2_ will be a 2D growth. With the supply of the sulfur vapor, the small islands grew into larger domain size, at last, the flat triangles of MoS_2_ were obtained, as shown in Fig. 4b. When the carrier gas flow rate is high enough, the chemical conversion occurs much faster than the diffusion of sulfur gas into the film. Under this condition, the sulfidation process will be rate-limiting. Meanwhile, with the anisotropic structure, the diffusion along the layers through van der Waals gaps is much faster than diffusion across the layers. Accordingly, the layers naturally orient perpendicular to the film, exposing van der Waals gaps for fast reaction. In this condition, the vertically structure formed, as shown in Fig. 4c.

**Figure 5:**
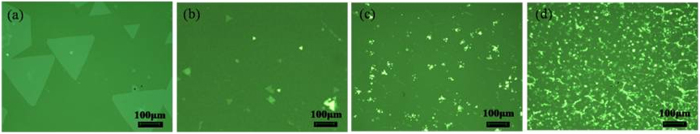
Optical microscope images of the synthesis MoS_2_ on Al_2_O_3_ with the carrier gas flow rate of 100sccm, 180sccm, 260sccm, 340sccm.

As we know, the wettability has a great effect to the nucleation process at the begining of the growth. If the wettability of the subbstrate is good, the film will be a two dimmendional growth, along the surface of the subbstrate, then flat film will be obtained. If the wettability of the subbstrate is poor, the film will maintain a 3D growth along the layers. In order to prove our conclusion, and have a contrast, we also synthesis the MoS_2_ film on sapphire with the same condition: the temperature of MoO_3_ powder is 1000 °C, carrier gas flow rate were 100sccm, 180sccm, 260sccm, 340sccm respectively. The results were shown in Fig. 5. we can see that, the film on the sapphire are all flat triangles. There is no vertically standing triangles obtained on the sapphire. This is because the perfect wettability of MoS_2_ on the sapphire, the growth process will be 2D. Under this condition, there will not be vertically standing triangles formed. In contrast, with a poor wettability between MoS_2_ and SiC, the nucleation of the film on the surface of SiC is more difficult, and the growth process will be 3D, which is useful to synthesis the vertically standing triangles. Through the results, we can see that the wettability is also a important factor during the growth of the vertically standing MoS_2_.

**Figure 6:**
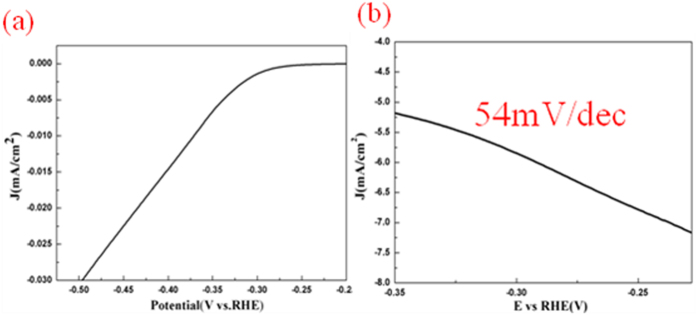
Cathodic polarization curves of vertically standing triangles, Tafel plots of vertically standing triangles.

HER catalytic activity of vertically standing MoS_2_ triangles on SiC was tested. Typical cathodic polarization curves and corresponding Tafel plots are shown in Fig. 6a,b. The Tafel slope in our vertically standing triangles was about 54 mV/decade. The related reports about vertically standing structures of MoS_2_ is about 94 mV/dec^10^ and 105–120 mV/dec^23^. Tafel plots are commonly used to evaluate the efficiency of the catalytic reaction. which indicated that the surface coverage of absorbed hydrogen was relatively low. The small Tafel plots means the high efficiency of the reaction. This indicating a good catalytic property of vertically standing triangles on SiC.

## Conclusions

We have developed a rapid sulfidation process for the synthesis of vertically standing MoS_2_ triangles. SEM images reveal the synthesis mechanism of the triangles. It is suggested that under a high concentration of sulfur, the growth process will by a 3D growth, all these vertically standing triangles come from the small islands on the surface of SiC. In addition, by a comparision between the films grown on sapphire and SiC, we find the wettability is another factor for the forming of vertically standing triangles. At last, the HER properties of the triangles was tested. The Tafel slope is about 54 mV/decade, which is much smaller than the related reports about vertically standing MoS_2_ nanosheets.

## Additional Information

**How to cite this article**: Lan, F. *et al*. Synthesis of Vertically Standing MoS_2_ Triangles on SiC. *Sci. Rep.* **6**, 31980; doi: 10.1038/srep31980 (2016).
